# HIV status disclosure during acute HIV infection in Malawi

**DOI:** 10.1371/journal.pone.0201265

**Published:** 2018-07-26

**Authors:** Sayaka Hino, Catherine Grodensky, Sarah E. Rutstein, Carol Golin, M. Kumi Smith, Lawrenson Christmas, William Miller, Sam Phiri, Cecilia Massa, Gift Kamanga, Audrey Pettifor

**Affiliations:** 1 Department of Medicine, University of North Carolina at Chapel Hill School of Medicine, Chapel Hill, NC, United States of America; 2 Center for AIDS Research, University of North Carolina at Chapel Hill, Chapel Hill, NC, United States of America; 3 Department of Health Policy and Management, University of North Carolina at Chapel Hill Gillings School of Global Public Health, Chapel Hill, NC, United States of America; 4 Department of Health Behavior, University of North Carolina at Chapel Hill Gillings School of Global Public Health, Chapel Hill, NC, United States of America; 5 Lighthouse Trust, Lilongwe, Malawi; 6 University of North Carolina Project Malawi, Lilongwe, Malawi; David Geffen School of Medicine at UCLA, UNITED STATES

## Abstract

Diagnosis of acute HIV infection (AHI) presents an opportunity to prevent HIV transmission during a highly infectious period. Disclosure is important during AHI as a means to facilitate safer sex practices and notify partners, particularly as those with AHI may be better able to identify the source of their infection because of the recency of HIV acquisition. However, little is known about disclosure during AHI. We conducted 40 semi-structured interviews with Malawians diagnosed with AHI (24 men; 21 married). Most participants reported disclosing to a sexual partner within a month of diagnosis, and knew or had a strong suspicion about the source of their infection. Participants often assumed their source had knowingly infected them, contributing to anger and feeling that disclosure is futile if the source already knew their HIV status. Assisted partner notification, individual and couples counseling, and couples HIV testing may facilitate disclosure during AHI.

**Clinical trial registration number:** NCT01450189.

## Introduction

Acute HIV infection (AHI) is a brief period immediately following infection characterized by extreme viremia in the absence of HIV-specific antibodies resulting in a highly infectious state and increased risk of transmission.[1–3] Because nearly all HIV testing worldwide uses antibody tests, current testing algorithms in resource-limited settings generally fail to identify persons with AHI. People with AHI are usually unaware that they have been infected and may spread the virus unknowingly. In generalized epidemic settings, such as Malawi, AHI may be responsible for almost 40% of new HIV transmissions,[4] although this rate may vary depending on the setting. Consequently, AHI presents an attractive target for early treatment and for prevention of onward transmission.

Self-disclosure of HIV status to sexual partners is an important strategy to prevent future transmission of HIV because it can reduce risky sexual behavior and improve HIV testing of sexual partners.[5, 6] Disclosure to partners may be particularly powerful during AHI for several reasons. First, given the high infectiousness in the AHI period, immediate risk reduction is urgent to protect others from acquiring HIV. Disclosing one’s status to current sex partners can mitigate the increased risk of AHI patients transmitting HIV during this period. Additionally, the recency of AHI patients’ infection may enable these individuals to infer the timeframe when they were infected, potentially improving their ability to identify the source of their infection and their ability to contact them. Notifying source partners creates an opportunity to prevent further transmission of HIV to other partners, especially if these partners are unaware of having HIV.

Significant challenges such as stigma and intimate partner violence have been shown to impede HIV disclosure attempts.[7, 8] However, little is known about patients’ particular challenges with disclosure to sex partners during AHI, their knowledge of who infected them with HIV, or their attitudes toward their source partners, who may conceivably be viewed unfavorably. In this study, we explore through qualitative interviews the barriers and facilitators of disclosure to sex partners among individuals diagnosed with AHI in Malawi and how knowledge of and attitudes toward the source of one’s HIV infection affects disclosure in the weeks following AHI diagnosis.

## Materials and methods

We report the results of a qualitative sub-study of the Methods for Prevention Packages Program (MP3) study, a randomized pilot study evaluating the potential public health benefits of behavioral and biomedical interventions for persons with AHI. In the MP3 study, men and women 18 years or older were recruited from two HIV testing and counseling centers and two sexually transmitted infection clinics in Lilongwe, Malawi. AHI diagnosis was defined as having a positive HIV RNA test and negative or discordant HIV antibody tests. Participants were excluded if they were diagnosed with established HIV infection (defined as two positive HIV antibody rapid tests), were HIV-negative, had a serious illness, had active drug or alcohol use that interfered with study requirements, or were imprisoned or voluntarily committed to the hospital for psychiatric or physical illness. Details of the MP3 study have been described elsewhere; briefly, participants were randomly assigned into a standard of care, motivational interviewing (MI), or MI plus 12-week antiretroviral therapy group.[9] The MI sessions focused on topics including knowledge of AHI, the importance of safer sex and partner notification, and disclosure.[9] The Biomedical Institutional Review Board of the University of North Carolina at Chapel Hill and the National Health Sciences Research Committee of Malawi approved this study, and informed consent was obtained from all participants.

As part of the qualitative sub-study, study staff conducted in-depth semi-structured interviews with participants four weeks after enrollment, three months after enrollment, and at their final study visit. This analysis focused on the week four interviews because of the proximity to infection and the specific line of questioning in the interview regarding participants’ source of infection. All interviews were conducted in Chichewa (the local language), audio-recorded, simultaneously translated and transcribed, and then verified by another staff member who compared the transcript to the audio-recording to ensure accuracy.

We conducted data analysis iteratively. Four members of the research team (SH, LC, MKS, CAG) read and wrote memos for interviews and then developed an initial codebook consisting of topical codes based on the semi-structured interview guides and inductive codes based on emerging themes from participant responses. After the codes were applied to randomly selected transcripts, the codebook was further refined, including defining code application more specifically, identifying additional themes that were not captured in the existing codes, and adding interpretive themes that applied across topical codes (e.g., stigma). To ensure consistency in coding, each team member coded at least five of the same transcripts as each other team member to facilitate familiarity with all members’ coding styles. All members of the research team coded every tenth interview, and team members met regularly to resolve discrepancies in their overlapping transcripts. These resolutions regarding code application decisions were documented on a spreadsheet shared by all team members to maintain uniform code application. Two research members generated and cleaned code reports, identified common themes, and created an analysis report for each parent code. These reports included themes for each child code (e.g., timing of disclosure, reason for disclosing), the number of participants expressing the theme, and a summary of the findings.

Participant responses to the question “Do you have a guess about who you got HIV from?” were used to categorize participants into three categories: “reports knowing source,” “suspects source but is uncertain,” and “reports not knowing source.” These categories were based on participant responses and not on researcher’s knowledge or interpretation of the situation. For instance, if a participant responded knowing with certainty who infected them despite mentioning several concurrent sexual partners around the time of infection, the participant was categorized as “Reports knowing source.” Participant disclosure to a source was determined by their responses to the question “Did you tell your sex partner(s) about your diagnosis with HIV?” followed by the question “Did you tell all of your recent sex partners about HIV?” when appropriate.

## Results

### Demographics and disclosure behavior

Forty participants, including 16 women and 24 men, completed a week four interview. About half (n = 21) of the participants were married at the time of the interview, and about half (n = 22) had not completed primary education. The average age of the participants was 28 years (range 18–51). Based on information collected during the interviews, having more than one sexual partner in the month before AHI diagnosis was more common among men than women. Most participants reported disclosing their serostatus to at least one sexual partner within a month of their diagnosis, although disclosing to all partners (both main and casual) was rare. Most (n = 14) of the participants who disclosed within the first month did so on the day of their diagnosis, but many (n = 9) also disclosed within the first one or two weeks. Finally, participants who did not disclose in the first month were mostly men and not married.

### Disclosure to current sexual partners

Other than two participants who reported that disclosure to their partner was accidental, all disclosed through direct communication with the partner. Most of those who had not yet disclosed to their current sex partner said they would like to be tested together as a couple.

The most common reasons participants reported for wanting to disclose to their partners were feelings of love and commitment to the partner, desire that the partner get tested, and simply feeling their partner should know. Common themes among all participants regarding reasons to *not* disclose to sexual partners were fears of their partners’ reactions, particularly the fear that partners would leave the relationship or tell others about their status (“*I am afraid that he will leave me… and also he might tell other people*, *everyone would know that I am HIV positive*”—Female, 21 years old). Participants also conveyed a desire to assess the seriousness and future of their relationship with a partner before deciding whether to disclose (“*I have to assess the future of the relationship before disclosure*. *I want to see first if we will end up marrying each other or not*. *If not then no need for disclosure*”—Male, 25 years old). Many participants said that they were less likely to disclose if the relationship was already over, and some said they were less likely to disclose if they had not really known or talked to a casual sex partner. Also, women seemed to view disclosure as their duty or responsibility as a wife, whereas men commonly assessed the nature of their relationship before deciding whether to disclose or not.

Characteristics of the sex partner also influenced participants’ disclosure. They seemed particularly unlikely to disclose to sex workers, whom they perceived as untrustworthy as well as difficult to locate due to their unstable living situations. One participant illustrated how partner characteristics, the future of the relationship, and partner trust combined to influence disclosure: “… *if I tell my partner*, *maybe she will not keep it a secret*. *Only the one that I know that we can marry and fulfill our goals together that can keep a secret for me [I will disclose to]*, *but this one is not trustworthy and therefore not fit to be told*” (Male, 24 years old). Additionally, some participants said they wanted to wait to disclose until their partner was in a “good mood”.

### Identifying suspected source of HIV infection

Overall, the majority of the participants reported knowing the source of their infection. Participants suspected partners to be the source of their infection for various reasons, including the time between a sexual encounter and the emergence of symptoms, having had unprotected sex with the partner, being monogamous, and having a history of previous negative HIV tests before sex with the partner. Other reasons participants suspected specific persons were more context-dependent and included interpersonal (rumors, distance in relationship) and environmental (community beliefs about HIV, traveling for work) factors that introduced risk to a relationship; these are described below.

Some participants discussed knowledge or beliefs about HIV that they used to inform their suspicions about the partners who may have infected them. The most common belief mentioned by participants was that persons with HIV were identifiable by their physical appearance; for example, participants pointed to a partner’s slimmer appearance or a “not happy, not healthy” body (Female, 24 years old) as evidence of HIV infection. Some participants were influenced by others’ impressions of their partners and cited rumors about their partners as reasons to suspect they had HIV. For example, when asked why she suspected her former husband as the source of her infection, one woman stated that she had been informed by neighbors in her home village that her husband had married another woman and that they were both taking ARVs. Similarly, to explain why he suspected one particular partner of having infected him among his four partners in the month prior to AHI diagnosis, one male participant stated that his friends told him that this partner’s previous husband fell ill and suspected that both she and her former husband had HIV. Most participants did not question the rumors they heard about their suspected sources, indicating the strong influence that peers and others’ impressions had on newly infected persons’ beliefs regarding the identify of their source of infection.

Participants also frequently recounted precipitating events that they thought had led to risky sexual behavior and their acquisition of HIV. Distance between partners, both geographic (e.g., travel or separate living situations) and emotional (e.g., separation in marriage), was common. One woman suspected her husband to be the source of her infection because he had been traveling for work and she had found text messages from other girlfriends when he returned. Several male participants discussed traveling in the month before their diagnosis and recounted instances that confirmed the increase in risky sexual behavior that occurred due to travel (e.g., “*When we are traveling*, *at times we are chatting at a beer drinking place*, *or not drinking but we are in a field elsewhere*, *it is possible at times that we fail to resist [sex]”—*Male, 25 years old). Two female participants described being separated from their male partners due to a temporary break in their relationship, and it was during this time when they lived apart that they suspected their partner had contracted HIV. These instances indicate a potential increase in HIV risk that can occur when partners are temporarily physically separated due to travel or a break in their relationship.

### Reaction to source information

Participants expressed a range of emotions towards their suspected sources of infection. Many of the reactions stemmed from the belief that their suspected sources were aware of their own HIV-positive status and had knowingly exposed the participant to risk of HIV. Some participants guessed that their source was aware of being HIV-positive because of how they behaved: “*Participant*: *She was actually refusing to come with me to the clinic*. *Interviewer*: *Do you think it was her first time to have the test*? *Participant*: *It seems she already knew her status*” (Male, 41 years old). However, most did not point to any specific behaviors that made them suspicious; they simply assumed the source knew his or her status and chose to not tell them: “… *if in the first place she did not disclose her status to me*, *it means that she had some ill thoughts*” (Male, 23 years old). Assuming the source partner was aware of being HIV-infected led some to feel deceived, even in cases when the suspected source’s HIV status was not verified. For some participants, feeling deceived led to anger that their source had knowingly infected them. Additionally, others felt disappointed and let down by their suspected source because of the perceived betrayal: “*For me to be found to be HIV positive because the woman who… I loved… I… I was not expecting that she could infect me with the illness which would finally have me tested for HIV*. *[Interviewer*: *participant is very emotional]*” (Male, 23 years old).

Interestingly, of the participants who suspected or knew their source, those who were in committed relationships (who were mostly women) frequently did not elaborate on their emotional reactions to the knowledge of their suspected source; rather, they focused on accepting what had happened and what to do given their diagnosis. “*I accepted [the diagnosis] because I love the man who infected me*. *Well*, *since he is my husband*, *I still need to accept it because if I desert him now that he is also HIV-positive; even if I leave*, *I would go elsewhere and then spread the infection*. *So I should just accept the way things are so that we should lead a careful life”* (Female, 23 years old)

### Disclosure to source

Most of the participants who reported suspecting or knowing the source of their infection stated that they disclosed to their suspected source shortly after their AHI diagnosis. Most of those who had already disclosed to their source partner identified the source as their spouse or main partner. Commitment to a relationship or marriage was the most common reason for disclosure to a suspected source. Although some reported the need to simply accept a committed source partner’s HIV status, others felt angry and betrayed at being infected, which presented a clear barrier to disclosure to source partners: *“You don’t disclose to somebody who didn’t disclose her status [to you]*, *because if she disclosed*, *I wouldn’t have gotten the virus*. *But she knew that she had the virus and on what we agreed in our relationship*, *I made love to her and she infected me with the virus*. *So I thought it wasn’t wise to communicate with such people*” (Male, 33 years old). Some participants felt wronged by their source and felt that their source had knowingly infected them, which affected whether and how disclosure occurred.

A sense of justice also manifested in participants’ desire for assurance that they would not be blamed for a partner having HIV if they disclosed. For example, when participants recognized that their source may not know that they had HIV, instead of feeling motivated to inform their source of their potential infection, they hesitated to disclose because they feared being blamed for the transmission of HIV. Many participants wanted to make sure that the source knew that the infection had come from themselves and not the participant. Concern that the source might not understand this directionality presented a barrier to disclosure. *“Aaaa…*.*to tell her that I am HIV positive… there can be friction a little bit because she may even think that I am the one who infected her and not vice-versa simply because it will be me to come out first and tell her of my HIV status*. *On the other hand*, *during the time we were doing sex*, *she never bothered to disclose her status* …*”* (Male, 24 years old). Participants seemed to want affirmation that they had been the victim or to ensure that it was clear that they had not been withholding their HIV status from their partner.

## Discussion

In general, patients newly diagnosed with AHI in Malawi struggled with the same reasons for and barriers to disclosure to sex partners as those demonstrated in the literature on chronic HIV infection.[10] For example, a systematic review of factors influencing HIV disclosure among people living with chronic HIV/AIDS infection in Nigeria identified the anticipated outcome of disclosure (e.g., partner reaction, fear of stigma and social exclusion), patient’s marital status, and knowledge of partners' HIV status as significant factors influencing HIV status disclosure; these factors were also reported in our study.[10] In our study, HIV disclosure had heightened significance due to the high level of infectiousness of the AHI period and the resulting urgency of adopting safer sex practices with current partners and the increased likelihood that AHI patients can identify and disclose to the source of their HIV infection.[11]

Because AHI diagnosis signifies a recent HIV transmission, this study presented a unique opportunity to explore the relationships between AHI patients and the sources of their infection and their particular facilitators and barriers of disclosure. In this study, most people diagnosed with AHI were able to identify or strongly suspect the source of their infection, relying on their knowledge of HIV, recollection of risky sexual behavior, assumptions or rumors about a partner’s HIV status, or conclusions based on physical or emotional distance with a partner. These findings about the recent life circumstances of acutely infected patients may suggest a particular need to target AHI testing to those who have recently been separated from or reunited with their partners, who may present to providers during an AHI period when they would test negative for HIV antibodies.

Despite being able to identify their source partners, AHI patients reported unique barriers to disclosure related to the perception that their partner infected them with HIV. For example, they frequently assumed that their suspected source was already aware of being HIV-positive and had knowingly infected them. This assumption discouraged open communication with source partners when participants felt deceived or angered by the person who infected them and when it seemed that disclosure would be futile (as there was no need to disclose to someone who already knew his or her HIV status). A study of AHI patients in South Africa also found that people diagnosed with AHI felt anger towards their suspected source of infection.[12] Although anger at source partners did not lead to any reported acts of violence in this study, verbal and physical abuse towards HIV-positive persons has been reported in several African countries including Malawi, South Africa, and Tanzania and may be a concern for those suspected of being the source of one’s HIV infection.[13]

Additionally, the nature of relationships clearly influenced how participants identified the suspected source of their infection and to whom they decided to disclose. Most of the participants who disclosed to a suspected source thought that their source was a spouse or a committed partner, supporting the idea that those with HIV are more likely to disclose to someone with whom they have or feel as though they have a committed relationship.[6, 14, 15] However, some participants in committed relationships also reported hesitating to disclose because they feared that their source partner would blame them for transmitting HIV. Given the urgency with which preventive behaviors and disclosure are needed during the highly infectious AHI period, both anger and blame present barriers that should be addressed with newly AHI-diagnosed individuals to reduce HIV transmission. These findings may indicate a need for increased individual counseling regarding the suspected source of one’s infection to help persons diagnosed with AHI cope with their anger and emotional reactions towards the source and initiate disclosure. Partner counseling, which has been shown to decrease blaming among partners for HIV infection,[16] may also help couples navigate these situations. Additionally, testing members of a couple for HIV/AHI at the same time may lessen the likelihood that the one who is diagnosed earlier will be blamed for infecting the partner who is diagnosed later.[17]

More than half of participants disclosed during the first month after they were diagnosed, with most of those disclosing on the first day and the rest within 1–2 weeks. It is likely that those who disclosed on the first day did not need any assistance to do so, possibly due to having regular contact and rapport with the partner to whom they disclosed. Most of those who disclosed after the first day but within the first 1–2 weeks were randomized to the intervention arm of the study, which delivered a counseling session focused on partner disclosure at about 1 week after diagnosis that may have supported their disclosure.[9] Those who did not disclose within the first month were more likely to be men and in casual relationships, and to anticipate negative reactions from their partners. Additional support such as assisted partner notification may be needed for this type of patient to manage the interpersonal and logistical barriers to disclosure.

In this study, participants specifically described weighing the future of their relationships and deciding to disclose to those to whom they felt committed. Participants described feeling less motivated to disclose to casual partners because they felt they may not see these partners again or that the casual partners knowingly infected them, particularly when the casual partners were sex workers. Notifying casual partners that they are at risk of acquiring or transmitting HIV is critical for improving early treatment and prevention efforts, particularly with sex workers who may have contact with multiple partners. Assisted partner notification may be a particularly important tool when AHI patients identify casual partners or sex workers as the source of their infection.[18] Involving third parties and other healthcare entities to notify partners that they have been exposed to HIV has been shown to be an effective strategy for identifying cases of undiagnosed HIV and providing earlier entry to care in the U.S., and this approach has recently been found to be feasible, but not widespread, in Sub-Saharan Africa.[19–21] A study in Malawi found that both contract referral, in which patients had seven days to notify their partner after which a provider did, and provider referral, in which the provider notified partners within 48 hours, resulted in significantly more partners receiving HIV testing and counseling than when patients self-disclosed.[21] Another study in Cameroon testing similar partner notification methods found similar results.[22] Assisted partner notification may thus be a beneficial and feasible strategy in the Sub-Saharan African setting to capitalize on the potential benefits associated with AHI testing while maintaining patient confidentiality.

This study has several limitations. We did not confirm the suspected sources of infection for participants whose partners were not referred and enrolled in the study, meaning we cannot say with certainty whether all of the individuals identified were in fact the source of their infection. However, some of these participants knew their partners had HIV and claimed they only had one partner in the month before diagnosis. Additionally, the study focused on participants’ experiences with a suspected source, not the accuracy of their identification, suggesting that the results regarding their experiences were valid. Furthermore, we used self-reported data on participant disclosure to sexual partners, which may have been subject to social desirability bias. Although the majority of participants did report disclosing to a partner, many of these individuals also reported not disclosing to other sexual partners, suggesting that the participants felt comfortable sharing when they had not disclosed. Finally, the participants were part of a larger parent study in which they had been randomized to receive MI with ART, MI alone, or standard care. Although the participants in the MI arms received more information about AHI and the importance of disclosure than those receiving standard care, in this qualitative sub-study, all of the participants were analyzed together.

## Conclusion

This study is the first we are aware of to explore in depth the views of acutely HIV-infected patients regarding disclosure of their HIV status to sex partners, including the person they suspect to have infected them. An AHI diagnosis confers several potential public health benefits over a diagnosis of established HIV, primarily the potential to interrupt the chain of HIV transmission while HIV is its most infectious. As persons diagnosed with AHI may have a better chance of identifying individuals known to be HIV-positive (i.e., the sources of their infection) due to the proximity of the diagnosis to acquisition, AHI diagnoses could lead to improved opportunities to notify the source and prevent further transmissions. However, disclosure during AHI has similar complications to disclosure at any point of infection. Our findings indicate that although many could identify the suspected source of their HIV infection, disclosure to these partners was hindered by feelings of anger, betrayal or being wronged, thus jeopardizing the potential benefits that could be gained from disclosure. Disclosure was also less likely among patients who felt less committed to their partners, who felt their partners were not trustworthy, and who felt their partners may blame them for introducing HIV into their relationship. The different barriers discussed during this study suggest that a variety of services may be required to enhance disclosure during AHI including assisted partner notification, individual and couples counseling, and couples HIV testing.
